# Lupus risk variants in the *PXK* locus alter B-cell receptor internalization

**DOI:** 10.3389/fgene.2014.00450

**Published:** 2015-01-08

**Authors:** Samuel E. Vaughn, Corinne Foley, Xiaoming Lu, Zubin H. Patel, Erin E. Zoller, Albert F. Magnusen, Adrienne H. Williams, Julie T. Ziegler, Mary E. Comeau, Miranda C. Marion, Stuart B. Glenn, Adam Adler, Nan Shen, Swapan Nath, Anne M. Stevens, Barry I. Freedman, Betty P. Tsao, Chaim O. Jacob, Diane L. Kamen, Elizabeth E. Brown, Gary S. Gilkeson, Graciela S. Alarcón, John D. Reveille, Juan-Manuel Anaya, Judith A. James, Kathy L. Moser, Lindsey A. Criswell, Luis M. Vilá, Marta E. Alarcón-Riquelme, Michelle Petri, R. Hal Scofield, Robert P. Kimberly, Rosalind Ramsey-Goldman, Young Binjoo, Jeongim Choi, Sang-Cheol Bae, Susan A. Boackle, Timothy J. Vyse, Joel M. Guthridge, Bahram Namjou, Patrick M. Gaffney, Carl D. Langefeld, Kenneth M. Kaufman, Jennifer A. Kelly, Isaac T. W. Harley, John B. Harley, Leah C. Kottyan

**Affiliations:** ^1^Immunology Graduate Program and Medical Scientist Training Program, University of Cincinnati College of MedicineCincinnati, OH, USA; ^2^Center for Autoimmune Genomics and Etiology, Cincinnati Children's Hospital Medical CenterCincinnati, OH, USA; ^3^Spelman CollegeAtlanta, GA, USA; ^4^Center for Public Health Genomics and the Department of Biostatistical Sciences, Wake Forest School of MedicineWinston-Salem, NC, USA; ^5^Arthritis and Clinical Immunology Research Program, Oklahoma Medical Research FoundationOklahoma City, OK, USA; ^6^Joint Molecular Rheumatology Laboratory of the Institute of Health Sciences and Shanghai Renji Hospital, Shanghai Jiao Tong University School of Medicine, Shanghai Institutes for Biological Sciences, and Chinese Academy of SciencesShanghai, China; ^7^Center for Immunity and Immunotherapies, Seattle Children's Research InstituteSeattle, WA, USA; ^8^Division of Rheumatology, Department of Pediatrics, University of WashingtonSeattle, WA, USA; ^9^Department of Internal Medicine, Section on Nephrology, Wake Forest School of MedicineWinston-Salem, NC, USA; ^10^Division of Rheumatology, Department of Medicine, David Geffen School of Medicine, University of California, Los AngelesLos Angeles, CA, USA; ^11^Department of Medicine, Keck School of Medicine, University of Southern CaliforniaLos Angeles, CA, USA; ^12^Division of Rheumatology, Medical University of South CarolinaCharleston, SC, USA; ^13^Department of Epidemiology, University of Alabama at BirminghamBirmingham, AL, USA; ^14^Department of Medicine, University of Alabama at BirminghamBirmingham, AL, USA; ^15^Rheumatology and Clinical Immunogenetics, University of Texas Health Science Center at HoustonHouston, TX, USA; ^16^Center for Autoimmune Disease Research, Universidad del RosarioBogota, Colombia; ^17^Department of Medicine, University of Oklahoma Health Sciences CenterOklahoma City, OK, USA; ^18^Rosalind Russell/Ephraim P Engleman Rheumatology Research Research Center, Department of Medicine, University of California, San FranciscoSan Francisco, CA, USA; ^19^Division of Rheumatology, Department of Medicine, University of Puerto Rico Medical Sciences CampusSan Juan, PR, USA; ^20^Center for Genomics and Oncological Research, Pfizer-University of Granada-Junta de AndaluciaGranada, Spain; ^21^Department of Medicine, Johns Hopkins University School of MedicineBaltimore, MD, USA; ^22^United States Department of Veterans Affairs Medical CenterOklahoma City, OK, USA; ^23^Division of Rheumatology, Feinberg School of Medicine, Northwestern UniversityChicago, IL, USA; ^24^Department of Rheumatology, Hanyang University Hospital for Rheumatic DiseasesSeoul, Korea; ^25^Division of Rheumatology, University of Colorado School of MedicineAurora, CO, USA; ^26^Divisions of Genetics and Molecular Medicine and Immunology, King's College LondonLondon, UK; ^27^United States Department of Veterans Affairs Medical CenterCincinnati, OH, USA

**Keywords:** lupus, PXK, fine-mapping, B cells, BCR

## Abstract

Genome wide association studies have identified variants in *PXK* that confer risk for humoral autoimmune diseases, including systemic lupus erythematosus (SLE or lupus), rheumatoid arthritis and more recently systemic sclerosis. While PXK is involved in trafficking of epidermal growth factor Receptor (EGFR) in COS-7 cells, mechanisms linking PXK to lupus pathophysiology have remained undefined. In an effort to uncover the mechanism at this locus that increases lupus-risk, we undertook a fine-mapping analysis in a large multi-ancestral study of lupus patients and controls. We define a large (257kb) common haplotype marking a single causal variant that confers lupus risk detected only in European ancestral populations and spans the promoter through the 3′ UTR of *PXK*. The strongest association was found at rs6445972 with *P* < 4.62 × 10^−10^, OR 0.81 (0.75–0.86). Using stepwise logistic regression analysis, we demonstrate that one signal drives the genetic association in the region. Bayesian analysis confirms our results, identifying a 95% credible set consisting of 172 variants spanning 202 kb. Functionally, we found that PXK operates on the B-cell antigen receptor (BCR); we confirmed that PXK influenced the rate of BCR internalization. Furthermore, we demonstrate that individuals carrying the risk haplotype exhibited a decreased rate of BCR internalization, a process known to impact B cell survival and cell fate. Taken together, these data define a new candidate mechanism for the genetic association of variants around *PXK* with lupus risk and highlight the regulation of intracellular trafficking as a genetically regulated pathway mediating human autoimmunity.

## Introduction

Systemic Lupus Erythematosus is the prototypical systemic autoimmune disease. Consequently, considerable effort has been devoted to understanding the genetic component of lupus risk. Now an understanding of causal mechanisms is approaching 10% of the 50 replicated genomic risk-loci that reach genome-wide significance (Vaughn et al., 2012).

Using a genome-wide association study (GWAS) design, we first identified association of a single nucleotide polymorphism (SNP) in the *PXK* locus with the occurrence of lupus in women of European descent (Harley et al., 2008). The *PXK* locus association with lupus-risk has since been replicated in several studies (Gateva et al., 2009; Suarez-Gestal et al., 2009). In these studies, the same variant identified in the initial GWAS was assessed in independent cohorts and the lupus-risk association was replicated (Gateva et al., 2009; Suarez-Gestal et al., 2009). These studies did not include any biological or functional genomic follow-up of the replicated association of this variant (Gateva et al., 2009; Suarez-Gestal et al., 2009). A fourth, recent study included a moderately-powered fine mapping analysis of a European cohort and used expression quantitative trait locus analysis of nearby genes to argue for a role of *ABHD6* in the increased lupus risk. For the current analysis, we initiated a well-powered fine-mapping study aimed at identifying the likely causal variants and defining the biological mechanisms of lupus risk at this locus.

PXK is part of the sorting nexin (SNX) family of proteins, which are important for receptor internalization, organelle trafficking including endosomal trafficking, and other membrane-centric sorting functions. This is accomplished primarily through the PX-domain mediated binding of PI_3_P (Xu et al., 2001; Seet and Hong, 2006). *PXK* was first identified and cloned by two independent groups in 2005 (Mao et al., 2005; Zou et al., 2005). Initial studies established that PXK is detectable in most tissues with a primarily cytoplasmic distribution. A more recent study in COS-7 cells demonstrated that PXK co-localized with the EGFR. Furthermore, PXK facilitated EGFR internalization following ligand binding, which was found to be PX-domain dependent (Takeuchi et al., 2010; Pedersen et al., 2012). *PXK* is widely expressed in the brain and blood (Zou et al., 2005), especially in B cells (Figure S1).

In this study, we identify a 257 kb region on chromosome 3, including all of *PXK* and a large region upstream of the gene that contains the lupus association signal. These results are confirmed via Bayesian analysis by which we identify a credible set consisting of 172 variants that explain 95% of the posterior probability in the region (Wellcome Trust Case Control Consortium et al., 2012). Through step-wise logistic regression analysis, we demonstrate that this region contains a single genetic effect.

Many studies in mice and humans highlight a central role of B cells in the etiology of lupus. Using the transcriptome of various B cell subsets, Hu et al. demonstrated that B cells and especially transitional B cells are enriched for transcripts near genetic variants associated with increased lupus risk (Hu et al., 2011). Autoreactive B-cells play a critical role in the development of autoantibodies that lead to immune complex deposition and lupus-associated tissue damage (Grimaldi et al., 2005). In murine studies, mice without B cells are largely protected from lupus-like disease (Shlomchik et al., 1994). Furthermore, murine studies established a clear role for B cells in the dysregulated cytokine production and T cell activation associated with lupus-like autoimmunity (Chan and Shlomchik, 1998; Chan et al., 1999).

B cell signaling through the B cell receptor (BCR) plays a critical role in the development of autoimmunity in lupus (Chaturvedi et al., 2011; Heesters et al., 2014). For example, BCR internalization facilitates Toll like receptor ligand internalization and signaling. Furthermore, type I IFN- a key cytokine known to play a role in lupus- is known to promote rapid BCR internalization (Chaturvedi et al., 2008; Giltiay et al., 2012). Based on its role in regulating EGFR, we hypothesized that PXK participates in BCR internalization and lupus-associated variants in the *PXK* locus differentially regulate BCR internalization. Indeed, we demonstrate that PXK colocalizes with the BCR and that the risk variants are associated with a decrease in BCR internalization. Knockdown of PXK replicates this phenotype, confirming the direct involvement of PXK in BCR trafficking.

## Methods

### Subjects and study design

We used a large collection of samples from case-control subjects from multiple ethnic groups. These samples were from the collaborative Large Lupus Association Study 2 (LLAS2) (Rasmussen et al., 2011) and were contributed by participating institutions in the United States, Asia, and Europe. According to genetic ancestry, subjects were grouped into ethnic groups including European American (EU), African American (AA), Asian and Asian American (AS), and Hispanic American (HA). Informed consent was obtained from all subjects using Institutional Regulatory Board approved consent documents. All lupus patients met the American College of Rheumatology (ACR) criteria for the classification of lupus (Hochberg, 1997).

### Genotyping of genetic variants and sample quality control

We genotyped 58 single-nucleotide polymorphisms (SNPs) covering the entire *PXK* region (58.1–58.5 MB on Chr 3, Build 37), as part of a larger custom genotyping study. The variants were chosen based upon the results of a candidate association study of 720 women of European ancestry and 2337 controls (Harley et al., 2008). Specifically, the variants were chosen to span the association interval identified with the Infinium HumanHap330 array. Genotyping of SNPs was completed with Infinium chemistry on an Illumina iSelect custom array according to the manufacturer's protocol. The following quality-control procedures were implemented to identify SNPs for analysis: well-defined clusters for genotype calling, call rate >90% across all samples genotyped, minor allele frequency (MAF) >1% (except for the rare variant analysis as described below), and *p* > 0.05 for differential missingness between cases and controls (the total proportion missing was <5%). One marker with evidence of a departure from Hardy-Weinberg proportion expectation (*p* < 0.0001 in controls) was removed from the initial analysis.

We removed individuals with a call rate <90% or excess heterozygosity. The remaining individuals were examined for excessive allele sharing as estimated by identity-by-descent (IBD). In sample pairs with excessive relatedness (IBD > 0.4), one individual was removed from the analysis on the basis of the following criteria: (1) remove the sample with the lower call rate, (2) remove the control and retain the case, (3) remove the male sample before the female sample, (4) remove the younger control before the older control, and (5) in a situation with two cases, remove the case with the less complete phenotype data available. Discrepancies between self-reported and genetically determined gender were evaluated.

### Ascertainment of population stratification

Genetic outliers from each ethnic and/or racial group were removed from further analysis as determined by principal component (PC) analysis and admixture estimates (Figure 1 of McKeigue et al., 2000; Price et al., 2006; Lessard et al., 2011). We used 347 ancestral informative markers (AIMs) from the same custom genotyping study that passed quality control in both EIGENSTRAT (Price et al., 2006) and ADMIXMAP (Hoggart et al., 2003, 2004) to distinguish the four continental ancestral populations, allowing identification of the substructure within the sample set (Smith et al., 2004; Halder et al., 2008). The AIMs were selected to distinguish four continental ancestral populations: Africans, Europeans, American Indians, and East Asians. We utilized principal components from EIGENSTRAT outputs to identify outliers of each of the first three PCs for the individual population clusters with visual inspection.

### Statistical analysis—workflow

The analysis was initiated by assessing the association of genotyped variants in each of the four ancestral cohorts individually as done previously at another locus (Kottyan et al., 2014). Strategically, we analyzed the genotyped, then imputed variants, performed full haplotype analysis, executed linkage disequilibrium analysis, and finally built a statistical model to account for the lupus-associated variability in European ancestry. In building the model of association in European Americans, we comparatively evaluated every variant in the region for its ability to better account for the lupus-associated genetic variation.

### Statistical analysis—frequentist approach

We tested each genetic variant for association with lupus using logistic regression models (frequentist approach) that included three admixture proportion estimates as covariates as implemented in PLINK v 1.07 (Marchini et al., 2007; Purcell et al., 2007). The additive genetic model is the primary model of inheritance. Other models were subsequently considered, but only if substantially superior. We performed a Cochran-Armitage trend test, a genotypic test, and tested both the dominant and recessive gene models. Logistic regression using an additive model remained the best model.

Step-wise logistic regression was performed to identify those genetic variants independently associated with the development of lupus in PLINK. For these analyses, the allelic dosage of a specific variant was added to the logistic model as covariates in addition to the admixture estimates. Haplotypic associations were assessed using logistic regression incorporating admixture measurements as covariates.

Linkage disequilibrium (LD) and haplotypes were determined with PLINK and HAPLOVIEW v 4.2 (Barrett et al., 2005; Sankararaman et al., 2008; Barrett, 2009). We calculated haplotype blocks for those haplotypes present at >3% frequency using the 4 gamete rule algorithms with a minimum *r*^2^-value of 0.8. Haplotypic associations were performed in PLINK using a 200 kb sliding window approach.

### Statistical analysis—bayesian approach

Using SNPTEST, we calculated the Bayes factor (BF) for each genetic variant: the probability of the genotype configuration at that genetic variant in cases and controls under the alternative hypothesis that the variant is associated with disease status divided by the probability of the genotype configuration at that variant in cases and controls under the null hypothesis that disease status is independent of genotype at that variant as previously described (we used the methods developed and introduced in Wellcome Trust Case Control Consortium et al., 2012). We used three admixture estimates as covariates, as we did for the frequentist approach. Large values of the Bayes factor (BF) correlate to robust evidence for association, as small *p*-values correlate to strong evidence in a frequentist approach. For our well-powered study, the Bayes factors (BFs) of the variants were highly correlated with the *p*-values (consistent with, Stephens and Balding, 2009). We used the additive model. The linear predictor is log(p_i_/(1-p_i_)) = μ + ßG_i_, and the prior is μ~N(0,1^2^), ß~N(0,0.2^2^) (variables are defined in the supplementary note in Wellcome Trust Case Control Consortium et al., 2012).

To identify the variants most likely to be driving the statistical association we calculated a posterior probability under the assumption that any of the variants within a single genetic effect could be causal and that only one of these variants is causal for each genetic effect. Variants with a low posterior probability are highly unlikely to be causal regardless of the allele frequency or presence of the actual causal variant in the analysis, following the procedure as presented (Wellcome Trust Case Control Consortium et al., 2012).

### Re-sequencing

We re-sequenced the *PXK* region as described previously (Lessard et al., 2012). DNA from European American subjects included in the current genotyping experiment was sequenced. To assess the accuracy of sequence-based SNP calling, we cross-referenced the sequenced and genotyped allele calls. Briefly, 3–5 micrograms of whole genomic DNA from each sample was sheared and prepared for sequencing with an Illumina Paired-End Genomic DNA Sample Prep Kit. Targeted regions of interest from each sample were then enriched with a SureSelect Target Enrichment System utilizing a custom-designed bait pool (Agilent Technologies). Post-sequence data were processed with Pipeline software v.1.7 (Illumina). All samples were sequenced to minimum average fold coverage of 253. Variant detection and quality control were also performed as previously described (Lessard et al., 2012).

### Imputation to composite 1000 genomes reference panel

To detect associated variants that were not directly genotyped, we imputed the *PXK* region with IMPUTE2 and using a composite imputation reference panel based on 1000 Genomes Project sequence data freezes from December 2013 (Marchini et al., 2007; Altshuler et al., 2010; Lofgren et al., 2010). Imputed genotypes were required to meet or exceed a probability threshold of 0.5, and information measure of >0.4, and the same quality-control criteria threshold described for the genotyped markers. In the statistical analyses, the probability threshold from each imputed value was incorporated into the statistical analysis using SNPTEST. The overall genotype-imputed variant concordance rate was >93%.

### Rare variant analysis

Rare variant analysis was performed in SVS Golden Helix SNP and Variation Suite v7.6.10 on the re-sequenced dataset and the imputed dataset. Rare variants were filtered based upon call rate and minor allele frequency <0.01. The one-sided kernel based adaptive cluster algorithm was performed using the hyper geometric kernel type with 1000 permutations in SVS GoldenHelix. For these studies, the initial analysis was performed using sequencing data from 92 lupus cases and 114 controls. Because this initial study was underpowered to identify an increased rare variant burden, we extended this analysis to an imputed dataset of all 4220 European lupus cases and 3803 European lupus controls. The accuracy of rare variant imputation, while limited, has been previously validated (Sung et al., 2012; Chen et al., 2013).

### Power analysis

Power analysis was performed using the Genetic Power Calculator (Purcell et al., 2003).

### Cell culture

Lymphoblastoid cell lines (LCLs) derived from control study participants without lupus were generated as previously described (Rasmussen et al., 2011) and maintained at 37°C in RPMI 1640 media containing 10% heat-inactivated fetal bovine serum (FBS) and antibiotic/antimicotic (Gibco/Life Technologies, Grand Island, NY). Peripheral blood mononucelar cells (PBMCs) were isolated using Ficoll-gradient centrifugation in SepMate tubes (Stemcell Technologies, Vancouver, BC, Canada) from fresh blood collected in acid citrate dextrose (ACD) tubes with informed consent from healthy donors. B cells were isolated using the B-cell isolation kit-II (Miltenyi Biotech, Auburn, CA).

### RNA purification and expression analysis

Total RNA was purified from LCLs using the RNeasy kit (Qiagen Valencia, CA). RNA was reverse-transcribed with the High-Capacity RNA-to-cDNA kit (Applied Biosystems, Grand Island, NY). Gene expression was determined by real-time PCR using a 7500 Real-time PCR system or Viia 7 system (Applied Biosystems). Relative quantification was calculated by the comparative CT method (Livak and Schmittgen, 2001). Briefly, expression levels for each target were normalized to levels of 18S ribosomal rRNA in the same well. All samples were then normalized to a selected sample and the data was exponentially transformed.

Protein expression was quantified in LCLs by flow cytometry. Cells were permeabilized and stained using reagents from Beckton, Dickinson and Company (BD, Franklin Lakes, NJ). Following staining, fluorescence was determined on an LSRFortessa analyzer (BD). The geometric median fluorescence intensity (gMFI) was calculated in FlowJo (Tree Star, Ashland, OR) and used as an estimate of protein expression. All experiments were run in triplicate. Statistical analysis and data visualization were done with GraphPad Prism (GraphPad Sftward, La Jolla, CA).

### BCR internalization and colocalization

BCR internalization was adopted from the method described by Malhotra et al. (2009a,b). Briefly, LCLs or freshly isolated PBMCs from subjects without lupus were incubated with anti-BCR for 30 min at 4°C. Cells were then placed at 37°C for the indicated times. Cross-linked BCR remaining on the surface was then detected with fluorescently labeled secondary antibody with the LSRFortessa Cell Analyzer. Percent internalization was obtained by measuring the gMFI at each time point and then calculating the change as follows: (gMFI no internalization (4° sample) – gMFI internalization (37° sample)) ÷ (gMFI no internalization (4° sample)) × 100. Statistical analysis was performed in R using the packages “nlme” and “lmerTest.” Cells were imaged with a confocal microscope and the ImageStreamX (Amnis, Seattle, WA) for co-localization analysis. All experiments were run in triplicate.

### shRNA transfections

shRNA were obtained from the CCHMC Lenti-shRNA Library Core, utilizing the Sigma Mission system. Viral vector DNA was isolated using Endofree Plasmid Maxi Prep kits (Qiagen). Lentiviral transduction was performed by the CCHMC Viral Vector Core. Viral particles containing the shRNAs were used to transfect LCLs from control subjects. Cells were plated onto retronectin-coated plates, viral particle supernatant added, and spun for 45 min at 1300 × G at 22°C. After resting at 37°C overnight, spinfection was repeated. Cells were transferred to selection media containing 2 ug/ml puromycin after 48 h. After non-transfected cells began to die (approximately 3–5 days) cells were tested for *PXK* expression and maintained for use in subsequent experiments.

### Materials

Anti-BCR (F(ab′)_2_ antibodies to IgG/IgM and Alexa Fluor 647-conjugated donkey anti-human IgG/IgM) were from Jackson ImmunoResearch (West Grove, PA). Anti-PXK antibody was purchased from Abcam (Cambridge, MA). Anti-LAMP1 antibody was purchased from BioLegend (San Diego, CA). Secondary antibodies were purchased from Life Technologies (Carlsbad, CA). Retronectin was purchased from Clonetech (Mountain View, CA). Puromycin was purchased from Invivogen (San Diego, CA). All other antibiotics and cell culture reagents were purchased from HyClone (Logan, UT) and Gibco (Life Technologies). Quantitative PCR was performed using TaqMan probes from Life Technologies.

## Results

**Figure 1:**
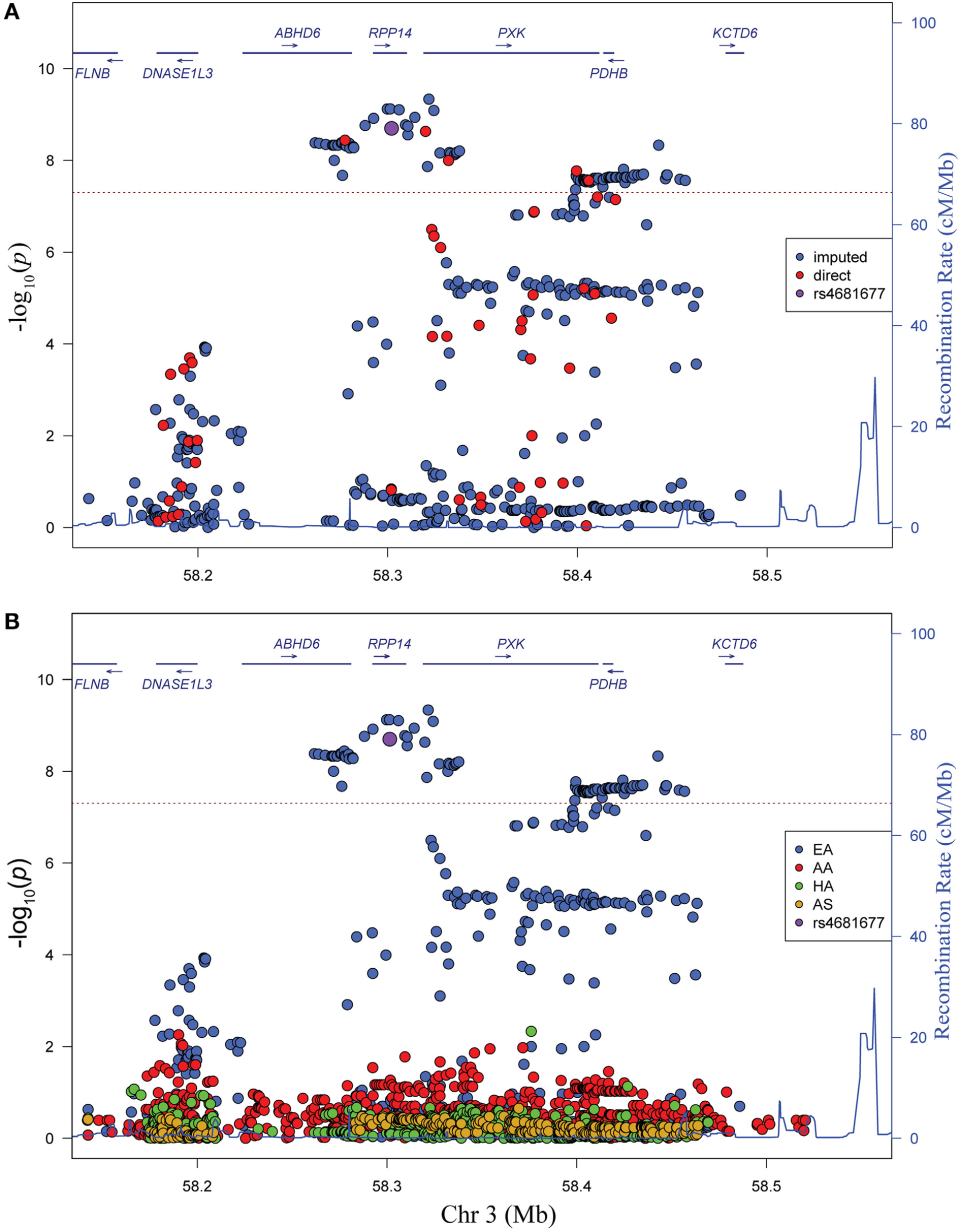
**Variants in the *PXK* locus are associated with lupus. (A)** Plot of –log_10_(*p*) values for directly genotyped (red) and imputed (blue) SNPs spanning the previously identified region of association with lupus. Gene locations are indicated above with arrows indicating direction of transcription. **(B)** Plot of –log_10_(*p*) values for different ancestral cohorts. EA, European ancestry; AA, African American ancestry; HA, Hispanic and Amerindian ancestry; AS, Asian ancestry. While not the most highly associated SNP, rs4681677 (purple) was used to choose samples for biological studies because it was genotyped; this SNP is in high LD (*r*^2^ > 0.9) with the imputed SNPs that are more highly associated.

We genotyped 57 useful markers from the *PXK* locus in a transancestral group of 18,286 lupus cases and controls. Our study included 8023 individuals of European descent (EA), 3740 individuals of African American descent (AA), 2481 individuals of Hispanic American descent (HA) and 2652 individuals of Asian descent (AS). To better capture the total variation in the locus, we imputed against a composite reference panel derived from the 1000 Genomes Project (Howie et al., 2011) for a final dataset of 269–835 variant markers, depending on ancestry, all with a minor allele frequency (MAF) greater than 0.01 (Table 1). We performed logistic regression analysis to reveal a large association region of 257 kb extending from just upstream of *PXK* beginning in *ABHD6* and extending through *RPP14* in individuals of European descent (Figure 1A). Overall, the most significant SNP was rs6445972 with *P* < 4.62 × 10^−10^, OR 0.81 (0.75–0.86). The most significant, directly genotyped SNP was rs4681677 with *P* < 2.00 × 10^−9^, OR 0.81 (0.76–0.87). Because rs4681677 was genotyped and not assessed solely through imputation, we used this SNP to classify cell lines as risk or protective for subsequent biological experiments. We found no evidence of the lupus-association at this locus in the other populations studied (Figure 1B). rs6445972 has an allele frequency of 30.0% in the EA cohort, 10.4% in the AA cohort, 22.9% in the HA cohort, and 0.2% in the AS cohort, suggesting that a largely European-derived allele is driving the very modest statistical association in the cohorts with European admixture.

**Table 1:** **Summary statistics for each study population**.

**Population**	**Case**	**Control**	**Male**	**Female**	**Genotyped**	**Imputed (MAF > 0.01)**
European (EA)	4220	3803	1696	6327	57	477
African American (AA)	1719	2021	766	2974	57	835
Hispanic (HA)	1599	882	244	2237	57	593
Asian (AS)	1310	1342	301	2351	57	269

*Cleaning steps are described in methods*.

**Figure 2:**
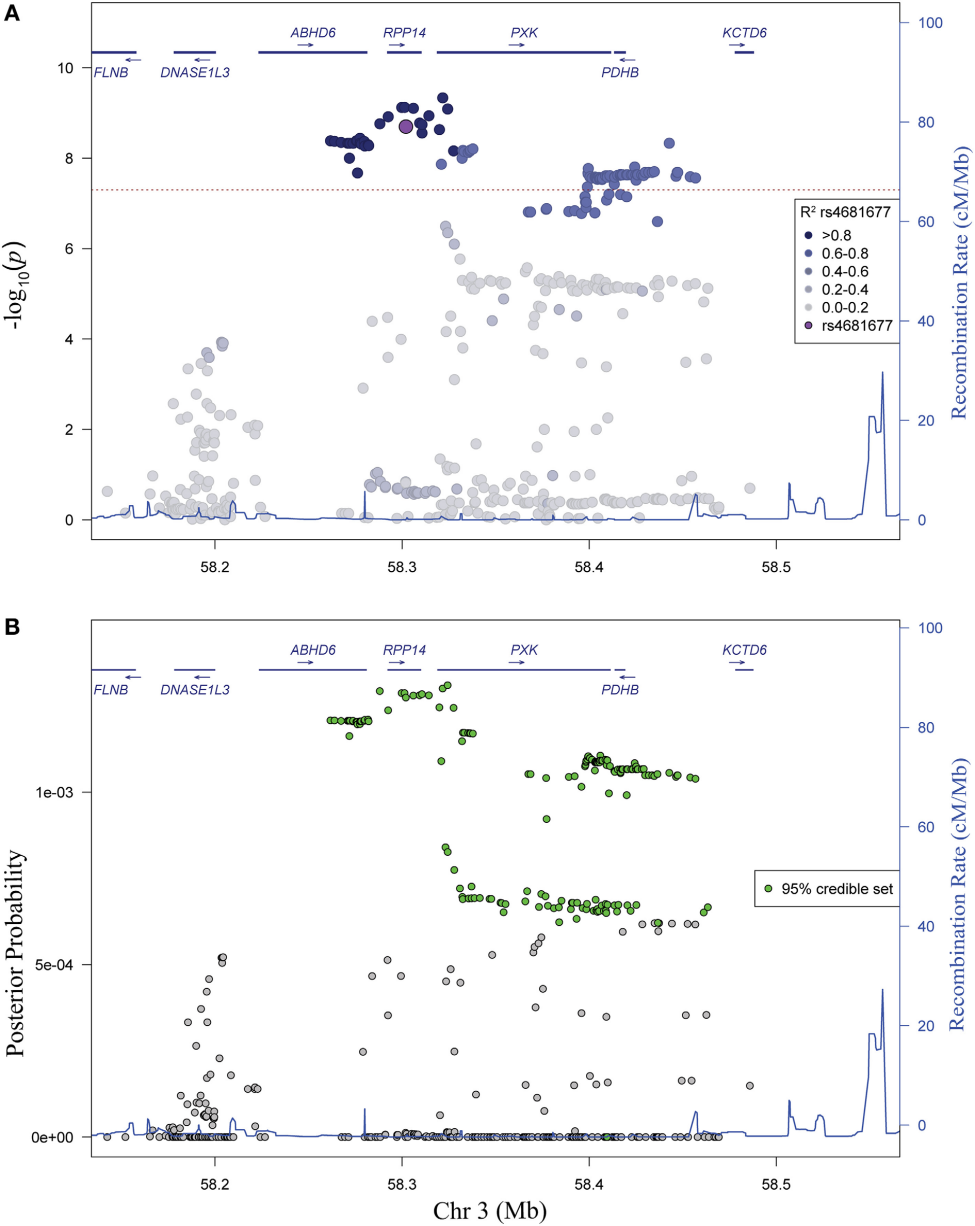
**Evaluation of linkage disequilibrium in the *PXK* locus and Bayesian association analysis. (A)** Association plot of genotyped and imputed SNPs shaded to identify the significant LD in the region. Colors correspond to *r*^2^-values, with darker shade indicating higher *r*^2^-values. **(B)** Bayesian analysis of variants in the *PXK* locus. Plot of posterior probability calculated for each variant in the locus. Variants included in the 95% credible set based on posterior probabilities are colored green.

Analysis of linkage disequilibrium (LD) in the region revealed high LD between the most strongly associated variants (Figure 2A). No single haplotype (using either blocks constructed from continuous groups of variants or the most highly associated variants) outperformed the single variant association model (data not shown), supporting the conclusion that the association in the region is due to a single genetic variant. In a complementary Bayesian analysis, we find a similar pattern of association, consistent with results from our frequentist logistic regression analysis. The 95% credible set (Wellcome Trust Case Control Consortium et al., 2012) contains 172 variants spanning a 202 kb region (chr 3: 58261741-58463411) (Figure 2B).

**Figure 3:**
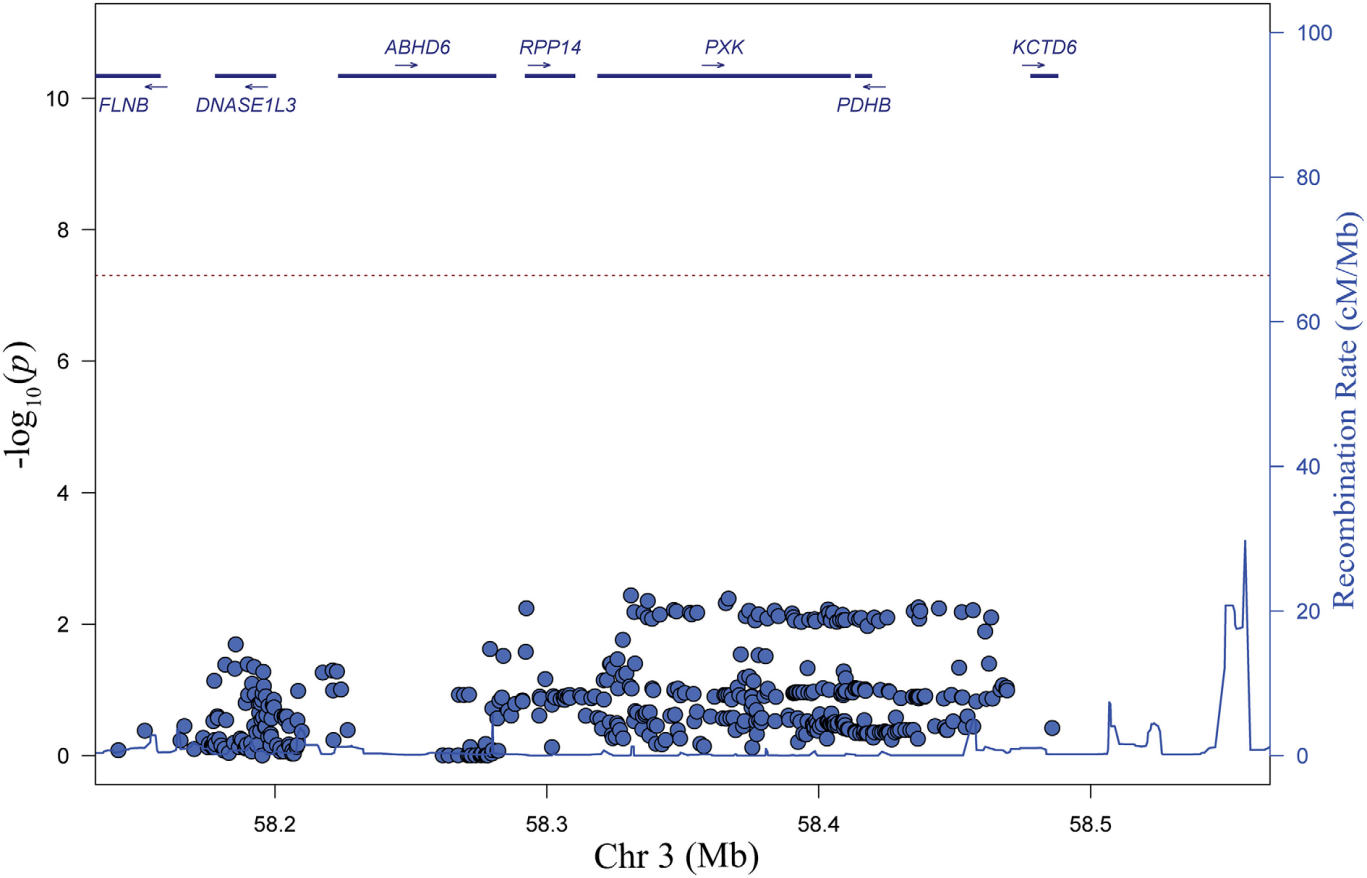
**Step-wise logistic regression analysis reveals one independent genetic effect at the *PXK* locus**. Plot of P values (–log_10_) following adjustment for genotype at rs4681677. Adjustment for other SNPs with *r*^2^ > 0.9 with rs4681677 can also account for the genetic association (*P* < 0.01) in the region.

To identify candidate variants in the EA population, we performed stepwise logistic regression to evaluate the ability of variants within the associated haplotype to explain all of the lupus-associated variation. Adjusting for any one of the top variants in the region eliminated the association signal, supporting the model that there is only one association in the region (Figure 3). We sequentially tested each variant in our dataset individually in our conditional analysis in an attempt to isolate groups of variants that may disproportionately carry the association signal in the region. While we did find clear groups, we found those variants that were able to adjust for the largest portion of the lupus-associated variation were in high LD (*r*^2^ > 0.8) with the most strongly associated variants. Notably, the SNP identified in our original GWAS study, rs6445975, did not account for lupus risk as completely as the most strongly associated variants in the current study (Figure 3 and Figure S2).

It remained possible that rare variants were driving the lupus association at the *PXK* locus. To test this possibility, we performed deep sequencing of the region in 92 cases and 114 controls of European ancestry. We found no statistical association of any of the variants with frequencies less than one percent using a logistic regression analysis with an additive model; furthermore, we found no increased burden of rare variants in any of the genes in the region in the cases compared to the controls (data not shown). Despite the limitations of imputed rare variants, we repeated the rare variant burden test on the complete European population and did not find evidence of increased rare variants in the lupus cases.

**Figure 4:**
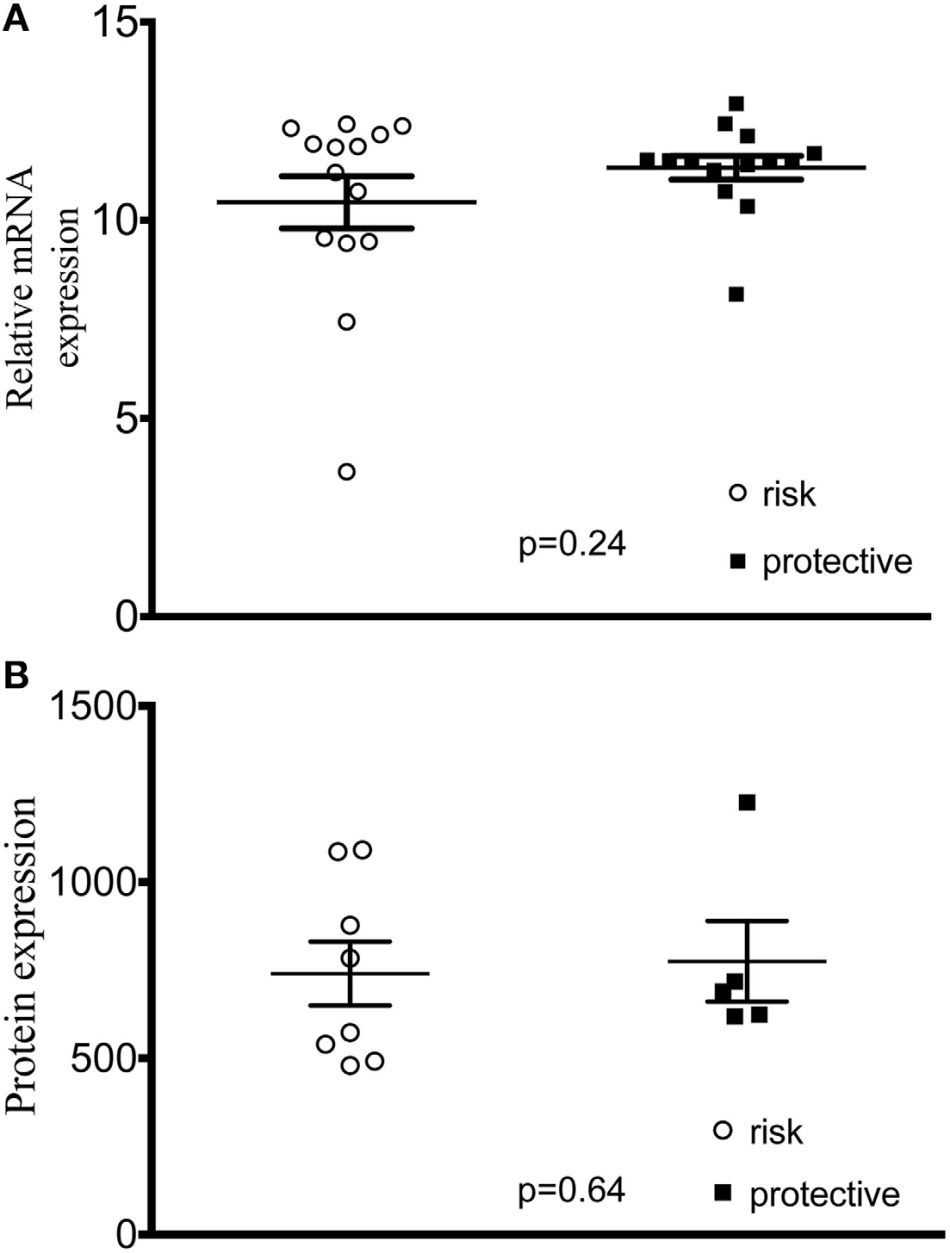
***PXK* expression in cell lines derived from study participants. (A)** Plot of ΔΔCt values of *PXK* mRNA expression using TaqMan probes. (*n* = 14 risk/14 protective) **(B)** PXK expression at the protein level using flow cytometetry. (*n* = 8 homozygous risk/5 homozygous protective). Data for both A and B are representative of 3 independent experiments using control cell lines. Statistical analysis using a Student's *t*-test was performed in PRISM.

In order to assess the hypothesis that lupus-associated variants were affecting the gene expression, we measured expression levels of all 5 genes in transformed B cell lines from control subjects and found no difference between cell lines with homozygous risk and homozygous non-risk genotypes (Figure S3). We did not find any difference in mRNA or total PXK protein expression (Figures 4A,B).

**Figure 5:**
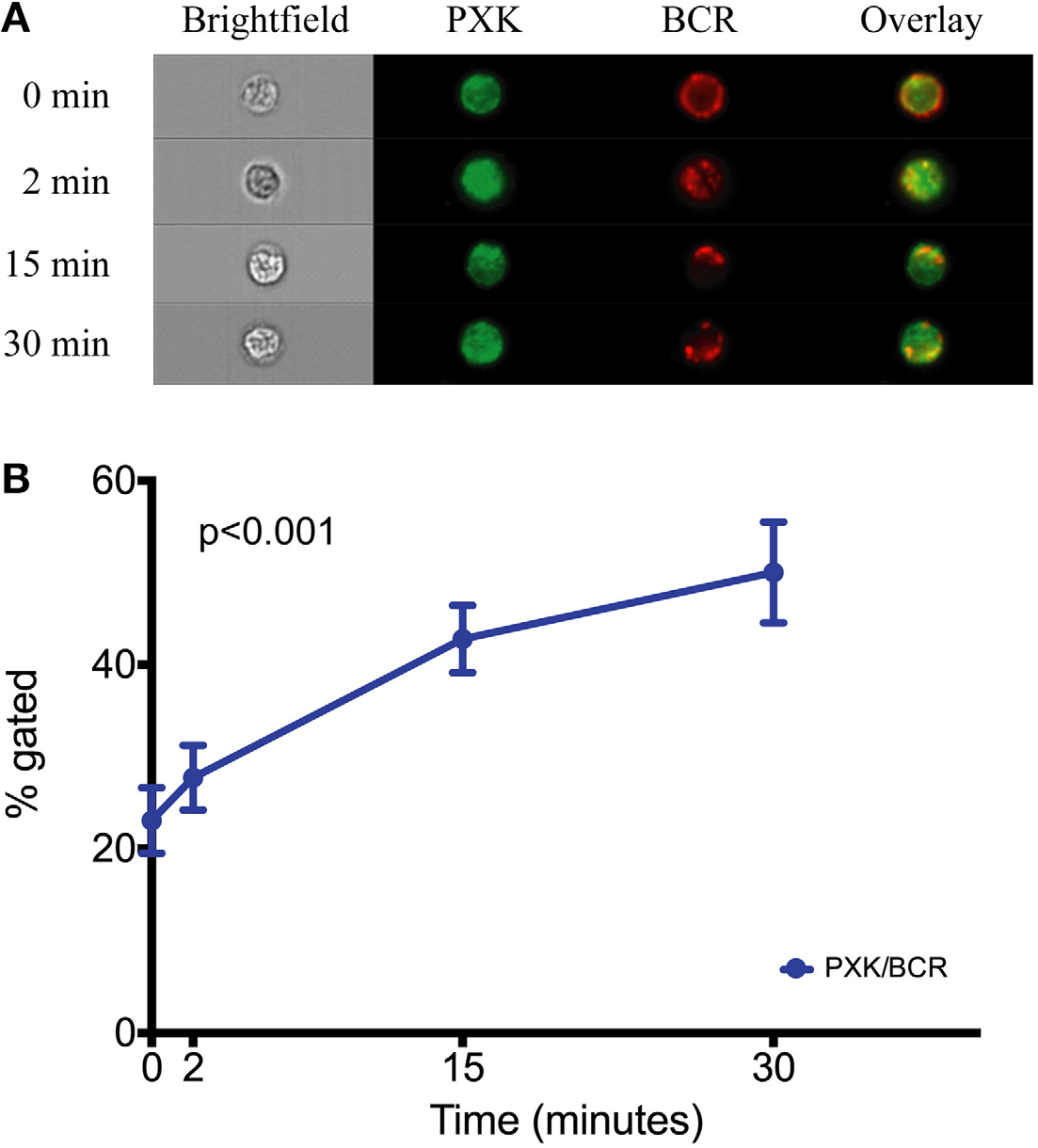
**PXK colocalizes with the BCR following receptor crosslinking. (A)** Quantification of colocalization between PXK and the BCR following BCR crosslinking in primary B cells from subjects without lupus. **(B)** Representative images showing colocalization. Statistical analysis using a One-Way ANOVA was performed in PRISM.

**Figure 6:**
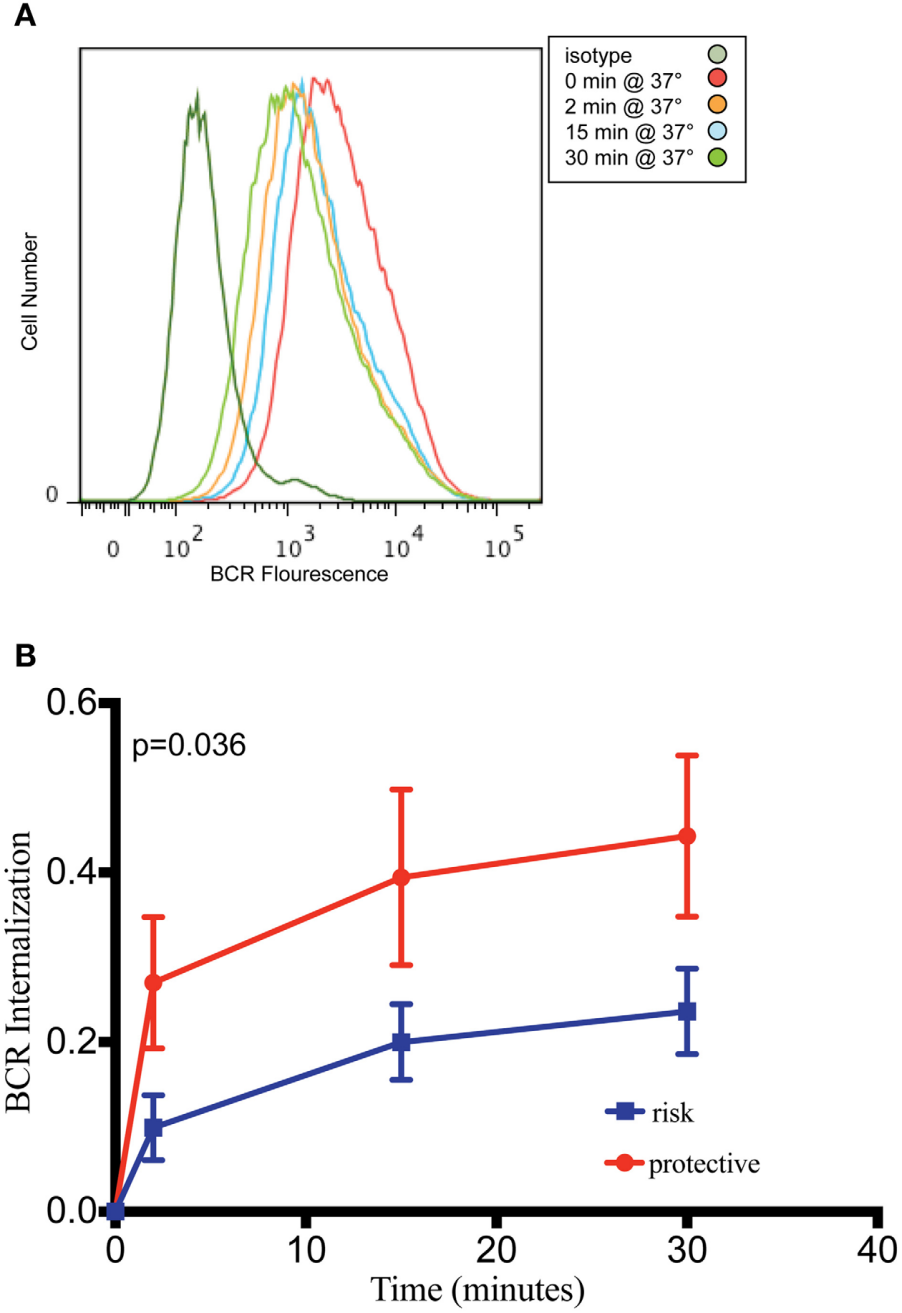
**B cells carrying *PXK* risk allele demonstrate decreased internalization of the BCR. (A)** Representative flow cytometry histogram showing decreasing fluorescence following internalization of the BCR from the B cell surface using patient-derived LCLs. **(B)** Quantification of BCR internalization as measured by the percent internalization (decrease in gMFI) following receptor crosslinking. Results of linear mixed model analysis as performed in R are shown. This experiment was performed using 20 cell lines from subjects without lupus in three independent experiments. The results of these independent experiments were combined and presented in **(B)**.

*PXK* is highly expressed in the B cell lineage, with the highest expression in mice observed in both transitional and follicular splenic B cells (Hu et al., 2011). The transitional B cell was recently implicated as one of the cell types critical to lupus pathogenesis based on a combined genetic and cell-specific expression analysis (Ramos et al., 2011). Given the expression of *PXK* in B cells, the fact that PXK has been shown to play a role regulating cell surface receptor expression, and the importance of B cells to the pathology of lupus, we hypothesized that PXK participates in the internalization of the BCR. We first evaluated colocalization between PXK and the BCR in B cells following BCR crosslinking on the cell surface. We found moderate steady-state colocalization between PXK and the BCR at baseline that increased following BCR crosslinking and continued to increase with cellular internalization (Figure 5). We then tested the hypothesis that the lupus-associated risk variants at the *PXK* locus affected the rate of BCR internalization by measuring the internalization of the BCR in cells derived from individuals with known genotypes. Following BCR crosslinking, we measured receptor internalization with flow cytometry and found that cells carrying the homozygous risk genotype displayed a decrease in the amount of BCR internalization compared to cells homozygous for the protective genotype (Figure 6). This finding was consistent across all the measurements over the time course of the experiment.

**Figure 7:**
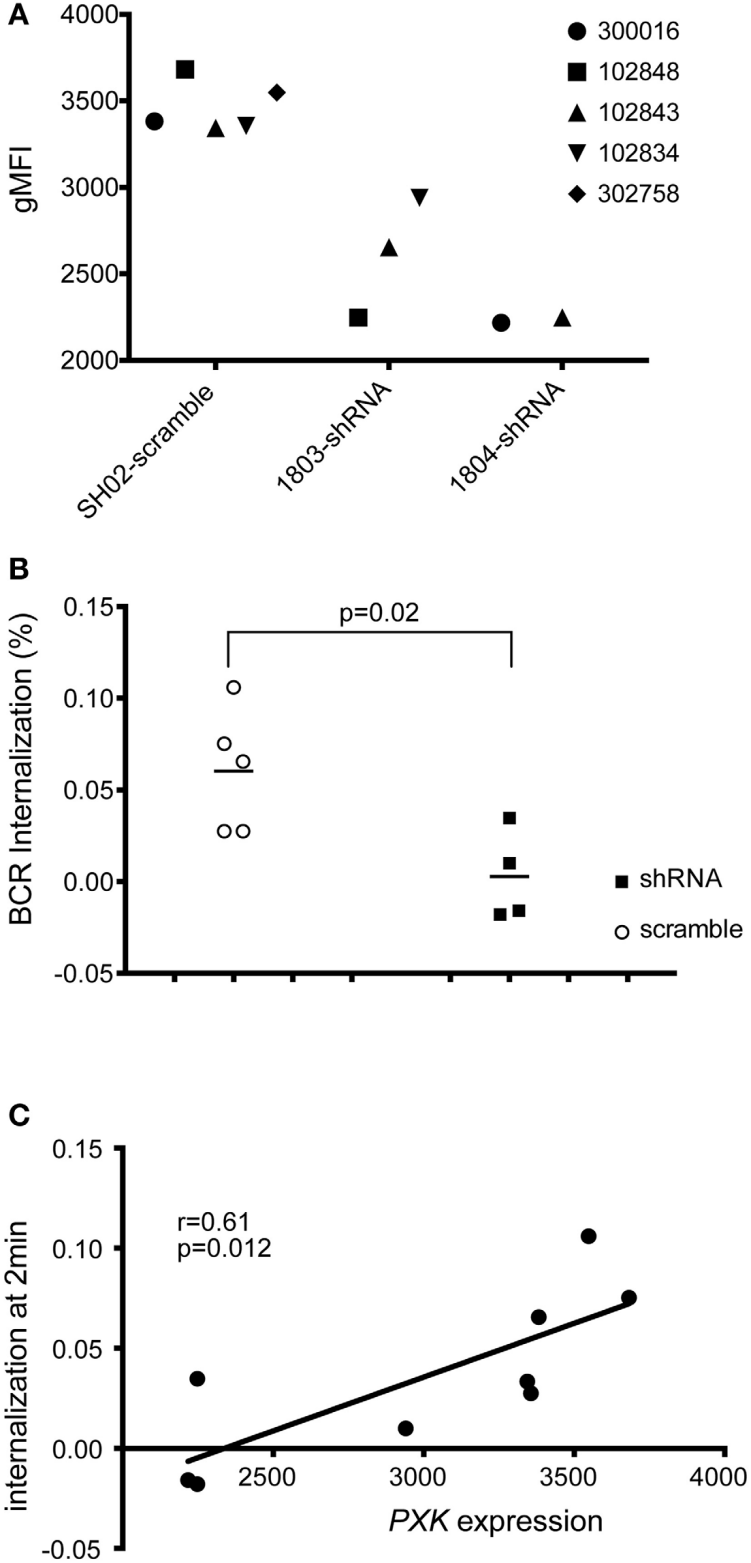
**shRNA knockdown of *PXK* disrupts BCR colocalization. (A)** PXK protein expression following shRNA knockdown in 5 LCL lines from non-lupus subjects (expression was assessed in triplicate for each cell line). **(B)** Summary of BCR internalization at 2 min in LCLs following viral transduction with either an shRNA against *PXK* or a scrambled control. Results from unpaired Student's *t*-test shown. **(C)** Correlation between PXK protein expression and BCR internalization following shRNA knockdown. The significance was assessed using a Pearson correlation. The graphs in **(B,C)** are representative of the results of three independent experiments.

To assess the role of PXK in the BCR internalization phenotype, we decreased *PXK* expression using shRNA by transfecting 5 study-derived cell lines from patient without lupus with shRNA targeted against *PXK* or scrambled controls (Figure 7A). Knockdown of PXK resulted in reduced internalization of the BCR, especially at the early time points (Figure 7B), and this reduction was directly correlated with *PXK* expression (Figure 7C).

## Discussion

We performed a fine-mapping study to define the genetic variants and the gene most likely to be causal for increased lupus risk. We genotyped 18,286 cases and controls from four ancestral populations, leveraging imputation to increase our coverage of the target locus. We used both logistic regression, and Bayesian methods to verify our findings that the disease risk from this locus is due to highly correlated variants in a large region including *PXK*. Additionally, we complemented our genetic fine-mapping with biological experiments, identifying a genotypic change in BCR internalization that is associated with the presence of lupus-risk variants.

Analysis of the *PXK* locus in non-European populations revealed no significant association in the region (Figure 1B). The allele frequency of the most highly associated variants differed amongst the ancestral groups, and given the observed allele frequencies, power analysis indicates that a much larger non-European population will be needed to detect a similar association of this magnitude at this locus (data not shown). Thus, the lack of an association in the other populations may be due entirely to the difference in power.

We found a 50% decrease in BCR internalization kinetics between risk and non-risk genotypes. This change in BCR internalization may have a small effect in the regulation of B cell signaling; however, it could also be meaningful in the context of other additive changes to the B cell signaling pathway (Vaughn et al., 2012). There are several mechanisms through which this functional change could affect the risk of autoimmunity. For example, stimuli that would be sufficient to develop strong B cell activation with subsequent negative selection may now be less likely to result in elimination due to attenuated BCR internalization. Indeed, the persistence of auto-reactive clones in the periphery may play an important role in the context of lupus pathogenesis.

PXK colocalized with the BCR and affected internalization, indicating a potential role for PXK in the regulation of BCR signaling. Importantly, the colocalization increased as the BCR is internalized into the cell, suggesting a role for PXK beyond the cell surface. Furthermore, we find that cells carrying the lupus-risk haplotype have a decreased amount of BCR internalization when compared to cell lines carrying the non-risk haplotype, an allelic functional change that we present as a candidate causal mechanism of increased lupus risk at this locus.

When *PXK* is specifically knocked down using shRNA, BCR internalization is decreased, confirming a clear role for PXK in regulating BCR internalization. These results support the conclusion that the allele-specific changes in BCR internalization we detect are most likely attributable to PXK. We do not yet understand the genetic mechanism behind this alteration in BCR internalization. We were unable to detect a difference in overall *PXK* expression. It may be that some other molecular differences are occurring after *PXK* expression, such as alternative splicing or differential post-translational modification. Future studies will be directed at detecting these changes. While *ABHD6* eQTLs were shown to correlate with lupus-association, there were also *PXK* eQTLs that were associated with lupus (Oparina et al., 2014). The *PXK* locus remains complicated and future work will be important to continue to unravel the specific genetic variations underlying the lupus association.

We and others refer to this locus as the “*PXK* locus” (Graham et al., 2008; Harley et al., 2008; Suarez-Gestal et al., 2009; Ramos et al., 2011; Oparina et al., 2014). Although our step-wise logistic regression analysis of common variants (Figure 3) makes it unlikely, it is still possible that there is a contribution of rare variant(s) that we missed. Of the 5 genes in the region, *PXK* and *DNase1l3* are the only genes with appreciable expression in immune cells based on evaluation of public databases (data not shown). *DNASE1L3* encodes the protein DNase 1-like 3, and is the only gene that has been investigated functionally in the context of lupus. A loss-of-function frameshift mutation in *DNASE1L3* was found to be associated with early-onset lupus and lack of detectable *DNASE1L3* transcripts in an autosomal recessive manner in six families of Arab descent (Al-Mayouf et al., 2011). *DNASE1L3* was also recently identified as a risk gene in systemic sclerosis (Mayes et al., 2014). The variant identified in that study shows no association with lupus in our dataset (*p* = 0.3067). *DNASE1L3* is removed from the peak association in the frequentist, logistic regression analysis and no members of the 95% credible set in the Bayesian analysis were located in or within 25 kb of *DNASE1L3*. It remains possible that variants many hundreds of killobases away from the promoter of a gene could affect that genes transcription (Westra et al., 2013); however, while both of our groups found detectable *DNASE1L3* transcripts, neither our group nor other groups have identified allelic expression of *DNASE1L3* in our cell lines (Oparina et al., 2014). Of the remaining genes, *ABHD6* has recently been suggested as the causal gene responsible for the association with lupus at this locus (Oparina et al., 2014). Mutations in *ABHD6* have been associated with multiple non-autoimmune phenotypes (Blankman et al., 2007; Alhouayek et al., 2013; Tchantchou and Zhang, 2013; Thomas et al., 2013; Volk et al., 2013), but beyond *ABHD6* being in the associated region there is no evidence, at present, for ABHD6 in lupus pathogenesis.

Overall, we identified a limited haplotype of highly associated variants in the promoter and first exon of *PXK* that account for all of the lupus-association in the region. We performed biochemical analysis to demonstrate that PXK co-localizes with the BCR and affects BCR internalization. Furthermore, we identified an allelic decrease in PXK-BCR co-localization and BCR internalization in subjects expressing the lupus-risk haplotype. Taken together, our work supports a model in which PXK increases lupus risk through the regulation of BCR internalization.

### Conflict of interest statement

The authors declare that the research was conducted in the absence of any commercial or financial relationships that could be construed as a potential conflict of interest.
