# Supplementary figures and images for: Lupus risk variants in the PXK locus alter B-cell receptor internalization
Source: Front Genet. 2015 Jan 8;5:450. doi: 10.3389/fgene.2014.00450 (PMC4288052; doi:10.3389/fgene.2014.00450)

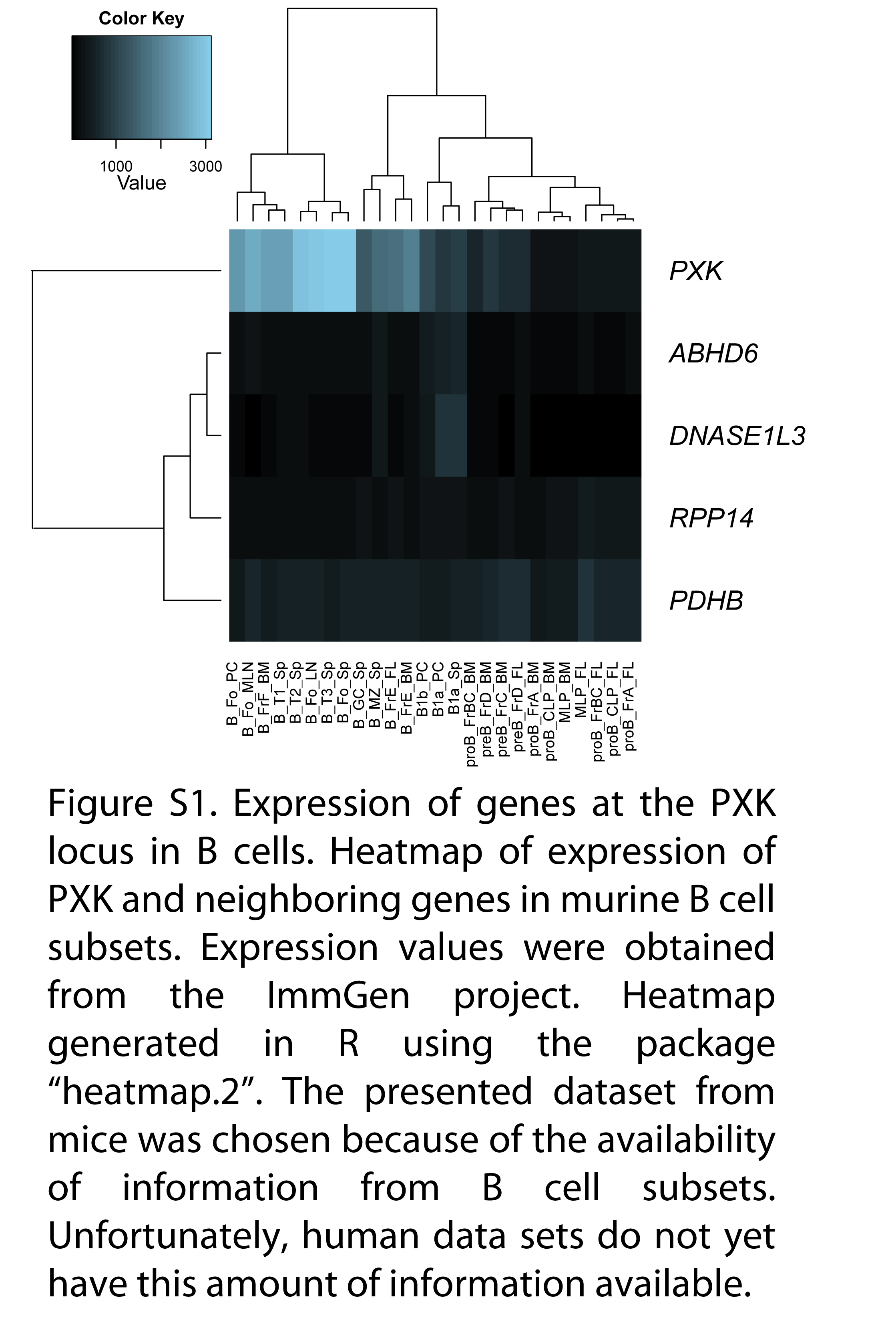

Supplement: Supplementary file 1 [file Image1.TIF]

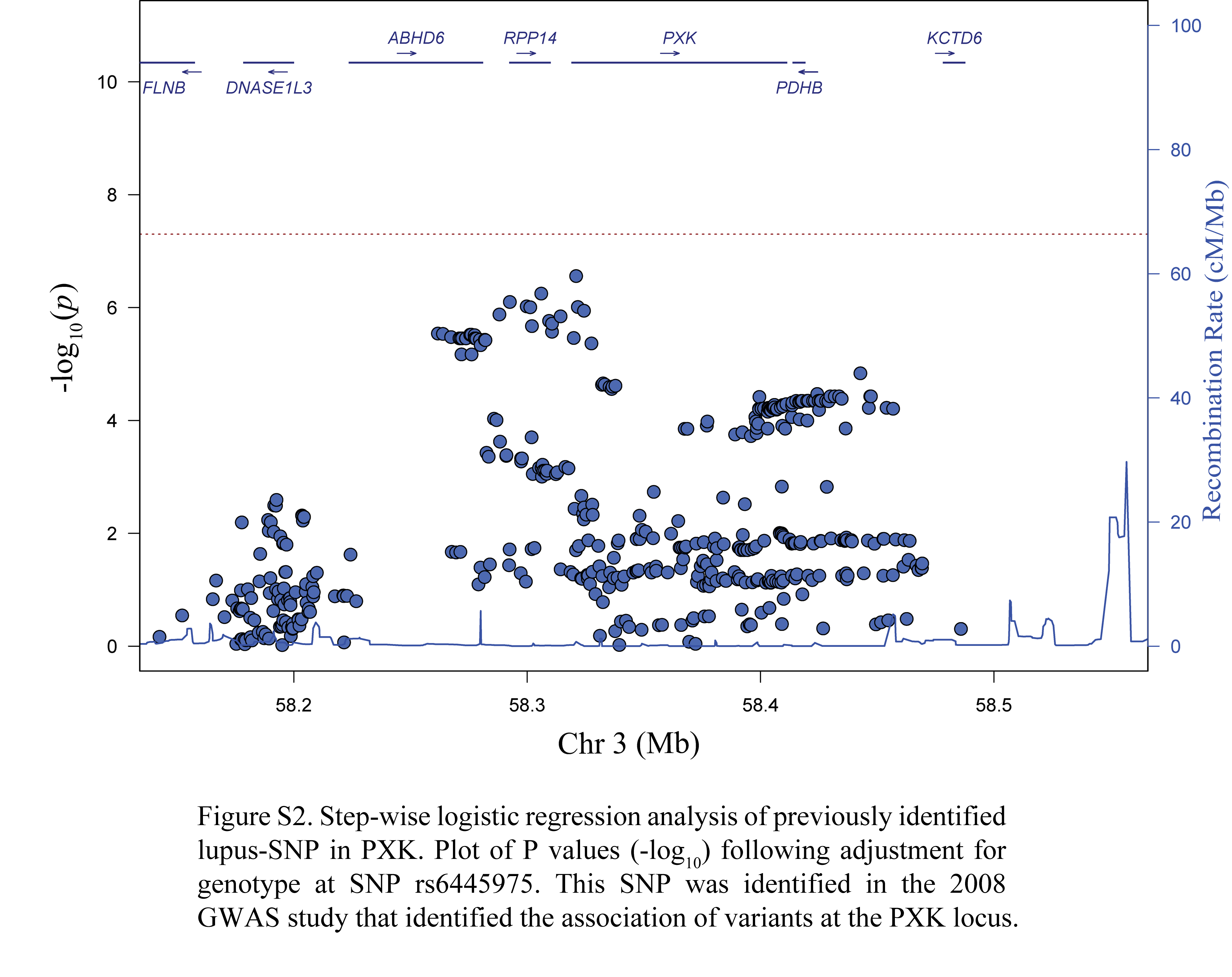

Supplement: Supplementary file 2 [file Image2.TIF]

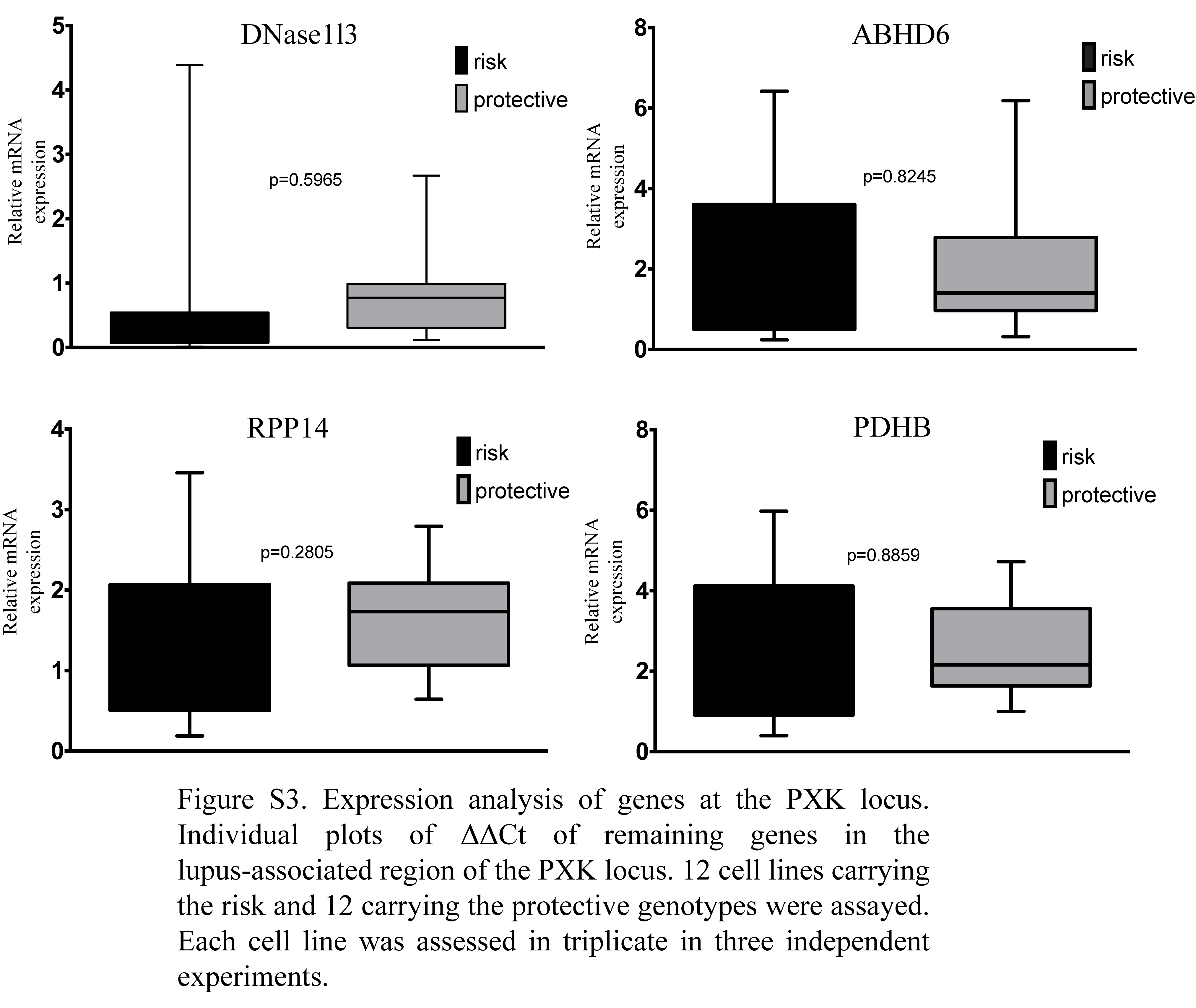

Supplement: Supplementary file 3 [file Image3.TIF]
